# Source time functions of earthquakes based on a stochastic differential equation

**DOI:** 10.1038/s41598-022-07873-2

**Published:** 2022-03-10

**Authors:** Shiro Hirano

**Affiliations:** Department of Physical Science, College of Science and Engineering, Ritsumeikan University, 1-1-1, Nojihigashi, Kusatsu, Shiga 525-8577 Japan

**Keywords:** Geophysics, Seismology

## Abstract

Source time functions are essential observable quantities in seismology; they have been investigated via kinematic inversion analyses and compiled into databases. Given the numerous available results, some empirical laws on source time functions have been established, even though they are complicated and fluctuated time series. Theoretically, stochastic differential equations, including a random variable and white noise, are suitable for modeling complicated phenomena. In this study, we model source time functions as the convolution of two stochastic processes (known as Bessel processes). We mathematically and numerically demonstrate that this convolution satisfies some of the empirical laws of source time functions, including non-negativity, finite duration, unimodality, a growth rate proportional to $t^3$, $\omega ^{-2}$-type spectra, and frequency distribution (i.e., the Gutenberg–Richter law). We interpret this convolution and speculate that the stress drop rate and fault impedance follow the same Bessel process.

## Introduction

Earthquake source time functions (STFs), which are temporal variations in the slip rate integrated over faults during earthquakes, are macroscopically observable in seismology and have been widely investigated regarding kinematic source inversions and dynamic source modeling. To review some knowledge on STFs, we first summarize some empirical laws (ELs) for STFs:

**EL1**: STFs are dominantly non-negative, continuous, compactly supported, and unimodal.
**EL2**: The moment functions $M_0$, which are proportional to the time-integration of STFs, evolve as $M_0 \propto t^3$, where *t* is the time since their ignition (this is referred to as “the cube law” herein).
**EL3**: The $\omega ^{-2}$-model can satisfactorily approximate the amplitude of STF Fourier spectra.
**EL4**: The frequency of their total moment follows the Gutenberg–Richter (GR) law.

Many studies, from early pioneering research^1^ to recent revelations^2,3^ have cataloged numerous STFs and revealed their tendencies and variabilities over time. Although several outliers have been found, EL1 has arisen as an obvious tendency, based on cataloged data. For example, $\sim$ 80% of STFs in a catalog^2^ are unimodal; they are labeled Group 1 in the research of Yin et al.^3^. In EL1, the fact that STFs are compactly supported is natural because regular earthquakes terminate within a few minutes, whereas slow earthquakes have longer durations.

Uchide and Ide^4^ compared the moment functions of $M_\text {w}$ 1.7–6.0 events in Parkfield, California, based on multi-scale inversion analyses. They pointed out that EL2 holds from the very early to later stages of the source processes. Meier et al.^5^ demonstrated that peak ground displacement evolves with the cube law. As the far-field ground displacement is proportional to STFs, they suggested that the law is sourced from the phenomenon of self-similar rupturing of the fault, which results in EL2. In addition, the proportionality between the final moment and the cube of the total duration has been established^1^. If the rupture duration is propotional to a fault length *L*, EL2 is equivalent to $M_0 \propto L^3$, which seems valid up to $M_\text {w}9$-class events on the basis of a global catalog^6^. However, traditional studies based on some regional catalogs have suggested scaling transitions to $M_0 \propto L^2$^7^ or $M_0 \propto L^1$^8^ for magnitudes greater than 7 or 8 possibly due to the thickness of seismogenic zones. Additionally, for $M_\text {w} \ge 7$ events, an analysis of observed STFs shows the $M_0 \propto t^2$ scaling^9^. Hence, we consider earthquakes below these magnitudes, where EL2 holds and the faults can be considered as finite 2-D planes smaller than the width of the seismogenic zone thickness.

Given the spectra of STFs, their amplitudes above their corner frequencies can be modeled by a power law, and their fall-off rates can be quantified. As shown by numerous studies^10–12^, EL3 seems to be very robust. Some forward modeling studies of dynamic rupturing have been conducted to explain the $\omega ^{-2}$-model; they have shown that STFs consist of functions that are almost entirely smooth, except for a kink. For example, Brune’s model has a kink at its start, while Sato and Hirasawa’s model and Madariaga’s model both have a kink due to their stopping phases^13^. Mathematically, these kinks are the origin of their spectral fall-off rate; put $(m, \mu )=(0, 1)$ in Theorem 1.2 of Nissilä^14^ for $\omega ^{-2}$. However, the cataloged STFs do not show such an isolated kink, but do show some fluctuations^3^. This implies that the traditional models as smooth curves with some finite number of kinks are too simplified to reproduce the complexity of STFs, and thus, that some stochastic modeling is required. The $\omega ^{-2}$-model with stochastic fluctuation has been modeled by using randomized phase spectrum^15^. However, such models required the duration of STF as an input, which should be determined stochastically to fulfill EL4.

Apart from the entire shape of each STF as discussed above, it has been well established that EL4 holds. The GR law originally means that the probability density function (PDF) of a seismic moment is a power law. By recalling the cube law between the moment and the duration, the GR law means that the PDF of the duration is also a power law. Once we model stochastic STFs, we can estimate the PDF of the duration and discuss whether the PDF satisfies the GR law.

The stochastic modeling of faulting processes has been proposed both theoretically and numerically^15–19^. Andrews^16,17^ considered a spatio-temporal slip distribution with self-affinity, mainly in the Fourier domain. This approach revealed the spectra of the distribution and energetics of the faulting. Significantly, the fault impedance, which is the factor of proportionality between the slip rate and stress drop in the Fourier domain, can enlighten the relationship between the quantities, even in the stochastic model. In recent, the importance of stochasticity has been more recognized. Spatial heterogeneity of fracture energy^18^ and temporal fluctuation of dynamic stress transfer^19^ introduced in a boundary integral equation play an important role on the rupture complexity. While such numerical modelings are developing, mathematical modeling, if available, would contribute to the understanding of complex faulting processes.

Stochastic differential equation (SDE)-based models have been employed in the field of earthquake source physics^20–22^. Matthews et al.^20^ and Ide^21^ modeled recurrent and slow earthquakes, respectively, as Brownian motion. On recurrent regular earthquakes^20^, they modeled a seismic cycle with a time scale longer than the characteristic time scales of each event, and the properties of STFs were not considered. Wu et al.^22^ assumed that the generalized Langevin equation can model the equation of motion for the fault slip rate. Although their model was based on some physical properties of dynamic friction, their solution was Brownian motion, which cannot satisfy the non-negativeness (EL1) or the $\omega ^{-2}$-like spectrum (EL3). Thus, a novel approach is needed for SDE-based modeling under EL1–4.

In this article, we consider an SDE known as the Bessel process. We analytically and numerically demonstrate that the convolution of two solutions from the same Bessel process satisfies EL1–4. Finally, we discuss the physical meaning of these two solutions on the basis of the fault impedance.

## Mathematical modeling

In the following, we do not distinguish STF $:= \displaystyle \int _\Gamma V(\varvec{x},t) \, d\varvec{x}$ and moment-rate function ${\dot{M}}(t) := \mu \displaystyle \int _\Gamma V(\varvec{x},t) \, d\varvec{x}$ on a flat fault $\Gamma$, where $\mu$ is the rigidity and *V* is the slip rate distribution at position $\varvec{x} \in \Gamma$. We introduce a mathematical model to generate ${\dot{M}}(t)$ that satisfies EL1–4 using solutions of an SDE. A Brownian motion well approximates ${\dot{M}}$ of slow earthquakes^21^ because the observed source spectra of slow earthquakes follow the $\omega ^{-1}$-model, which is similar to the spectrum of Brownian motion. However, because EL3 holds for regular earthquakes, we consider a product of the spectra of two stochastic processes (i.e., $\omega ^{-1} \times \omega ^{-1} = \omega ^{-2}$), which is a convolution of the two stochastic processes in the time domain, which we denote as $X_t^{(1)}$ and $X_t^{(2)}$ herein. Thus, we assume that ${\dot{M}}(t) = \left( X_\bullet ^{(1)} * X_\bullet ^{(2)}\right) (t)$ holds, where the asterisk “*” denotes the convolution in time.

To fulfill EL1, we assume that both $X_t^{(1)}$ and $X_t^{(2)}$ are solutions of the following SDE called the Bessel process:

1 $$\begin{aligned} dX_t^{(i)} = \frac{d-1}{2} \frac{dt}{X_t^{(i)}} + dB^{(i)}_t, \quad (i=1, 2) \end{aligned}$$

with its initial value $X_0^{(i)}$ ($> 0$), which is equivalent to the integral form as:

2 $$\begin{aligned} X_t^{(i)} = X_0^{(i)} + B_t^{(i)} + \frac{d-1}{2} \int _0^t \frac{ds}{X_s^{(i)}}, \quad (i=1, 2) \end{aligned}$$

where $B_t^{(i)}$ is a standard Brownian motion and *d* is the dimension of the Bessel process. SDE(1) is valid while $X_t^{(i)}>0$ holds. Thus, we define $X_t^{(i)}=0$ after the process hits zero; the time $T := \displaystyle \min_t \left\{ t \ | \ t > 0 \, \& \, X_t^{(i)}=0 \right\}$ is referred to herein as the first hitting time^23^. According to the above definition, $X_t^{(i)}$ is continuous and non-negative. Moreover, $X_t^{(i)}$ with $d<2$ is compactly supported because $T \ll \infty$ holds almost surely for that parameter range^23^. Therefore, given $d < 2$, EL1 holds if we can confirm that the convolution is unimodal. We demonstrate this statement numerically in the next section.

We also confirm that the convolution satisfies EL2 and EL3 numerically in the next section. It can be expected that EL3 would be satisfied, as described in the first paragraph of this section.

**Figure 1:**
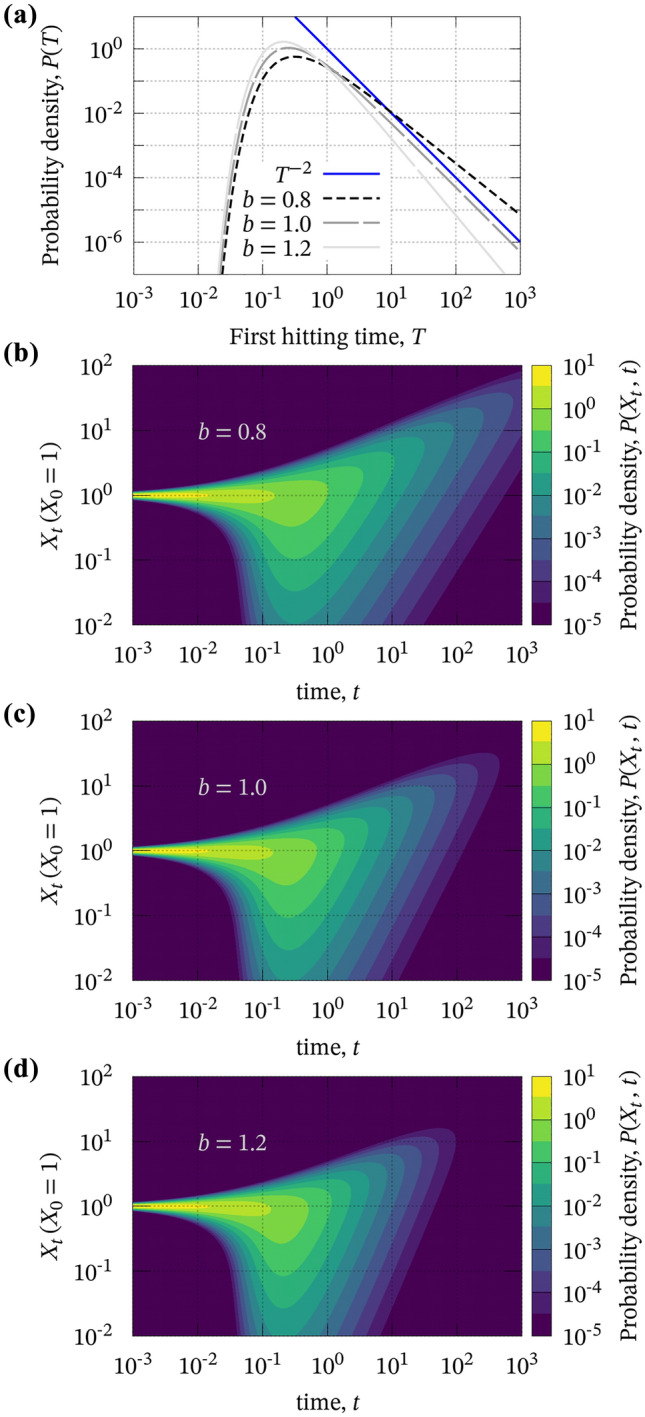
Probability density functions of (**a**) the first hitting time for $b=$ 0.8–1.2 based on Eq. (3) and (**b**)–(**d**) $X_t$ for $b = 0.8$–1.2 based on a solution of the Fokker–Planck equation^25^, where *b* is the *b*-value of the GR law and related to the parameters as in Eq. (5). We assume $X_0 = 1$ for all figures. The blue line in (**a**) represents $T^{-2}$ (Eq. (4) with $b=1.0$) and is parallel to the dashed curve (Eq. (3) with $b=1.0$).

The condition for EL4 can be derived analytically. It is known that *P*(*T*), which is the PDF of the first hitting time *T* with $d < 2$ and $X_0^{(i)} = a$, can be represented as^24^:

3 $$\begin{aligned} P(T) = \frac{2^\nu }{a^{2 \nu } \Gamma (|\nu |)} T^{\nu -1} \exp \left( -\frac{a^2}{2 T}\right) , \end{aligned}$$

where $\nu = \displaystyle \frac{d}{2}-1$, and $\Gamma (\cdot )$ is a gamma function. On the other hand, considering the cube law ($M_0 \sim T^3$), the GR law with respect to $M_{\textrm{w}} = \frac{2}{3} \log _{10}{M_0}-6.1$ can be represented as

4 $$\begin{aligned} P(M_{\textrm{w}}) \sim 10^{-b M_{\textrm{w}}} \sim T^{-2 b}, \end{aligned}$$

where $b \sim 1$ holds and the constant coefficients are neglected. Thus, if we assume a sufficiently small initial value, $a \, (\ll \sqrt{2 T})$, Eqs. (3) and (4) imply that:

5 $$\begin{aligned} \nu = -2 b + 1, \quad \text {i.e.,} \quad d = 4 ( 1 - b) \end{aligned}$$

is required for EL4. By combining Eqs. (3) and (5), we show the PDF of the first hitting time with $b=0.8$–1.2 in Fig. 1a. On the other hand, $1-P(T)$ represents the probability that the random variable $X_t$ does not fall into zero at each time. Within the probability, the probability density of $X_t (>0)$ for each *t* has been known as a solution to the Fokker–Planck equation accompanied by Eq. (1)^25^; Fig. 1b–d show the solution that indicates a probability of evolution of $X_t$. We speculate about a physical interpretation of this probability in the discussion section.

## Numerical modeling and results

In the following section, we investigate how the convolution $\left( X_\bullet ^{(1)} * X_\bullet ^{(2)}\right) (t)$ satisfies EL1–3 after solving Eq. (1) using the SRIW1 algorithm^26^ implemented in DifferentialEquations.jl (https://diffeq.sciml.ai/) for Julia 1.6.1 (https://julialang.org/); the associated function and numerical results in an ascii format are available at a repository (https://doi.org/10.17605/OSF.IO/VUXJ6). Given Eq. (5) and $b = 1$, we solve:

$$\begin{aligned} dX_t = - \frac{1}{2} \frac{dt}{X_t} + dB_t \end{aligned}$$

with a constant time step of $dt = 10^{-6}$ and a sufficiently small initial value of $X_0 = 10^{-3}$ up to time $T_\text {max} = 2 \times 10^{-3}$ (i.e., 2000 steps). Because the solution must become zero within the finite time, we reject numerical solutions that never reached zero before $T_\text {max}$. The convolution of two solutions does not follow $\omega ^{-2}$-model if their corner frequencies, which are comparable to the inverse of their first hitting time, are quite different. Thus, we denote the lower limit of the first hitting time as $T_\text {min}$ and reject solutions that reach zero before $T_\text {min}$. In the following, we investigate two cases: A) $T_\text {min} = 1 \times 10^{-3}$ (i.e., 1000 steps) and B) $T_\text {min} = 2 \times 10^{-4}$ (i.e., 200 steps). Therefore, we consider the Bessel processes with the probabilistic first hitting time *T* satisfying $T_\text {min} \le T \le T_\text {max}$, where $T_\text {min}/T_\text {max} = 0.5$ for case A and $T_\text {min}/T_\text {max} = 0.1$ for case B. For every two solutions, we regard the solution with relatively shorter duration as $X_t^{(1)}$ and the other as $X_t^{(2)}$. Thus, $T_\text {min}/T_\text {max} \le T_1/T_2 \le 1$ holds, where $T_i$ is the duration for $X_t^{(i)}$ ($i=1,2$).

After iterations, we store 2000 solutions with $T_\text {min} \le T \le T_\text {max}$, which yields 1000 pairs of solutions, and calculate 1000 convolutions of the pairs. Even though we calculate and abandon many useless solutions, we obtain $\sim$ 120 Bessel processes per minute within the duration range by using 12-core AMD Ryzen 9 3900XT.

**Figure 2:**
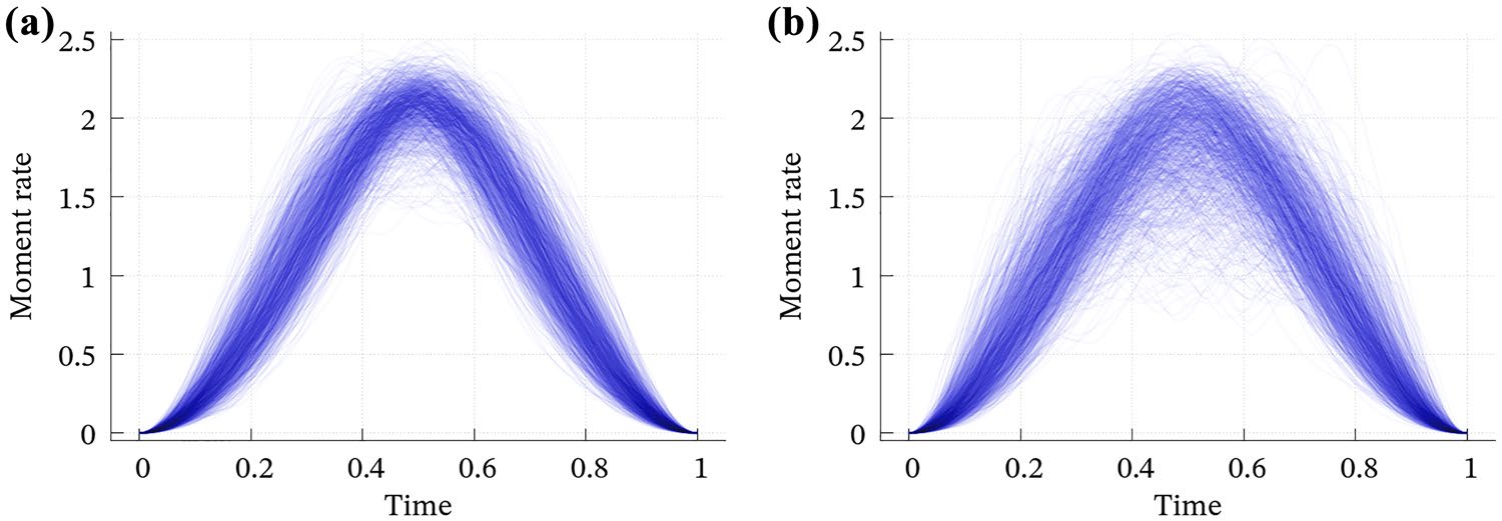
The 1000 computed convolutions of the two Bessel processes for (**a**) case A and (**b**) case B. Time scale and total moment are normalized. See Figures S.1 and S.2 for individual curves.

**Figure 3:**
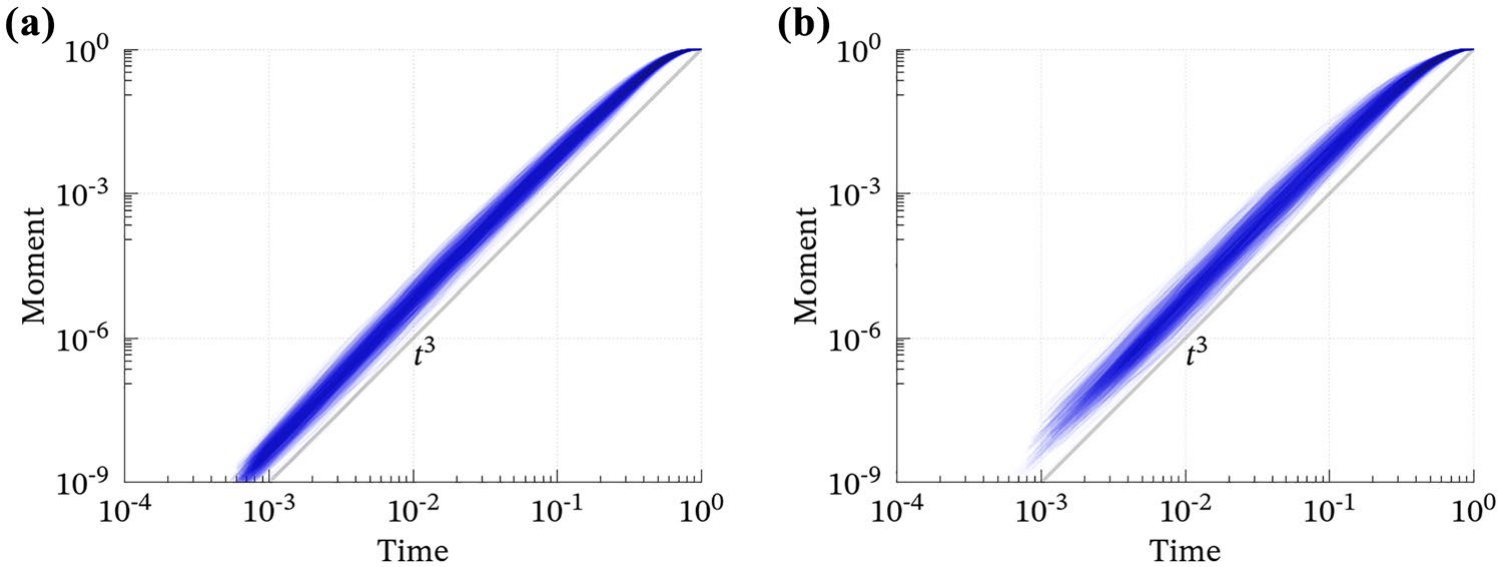
The normalized moment evolution $\left( \displaystyle \int_0^t {\dot{M}}(s) \, ds / \displaystyle \int _0^\infty {\dot{M}}(s) \, ds \right)$ for (**a**) case A and (**b**) case B along normalized time scale (*t*/*T*). The curves dominantly follow the cube law ($\sim t^3$) and converge toward their static states.

**Figure 4:**
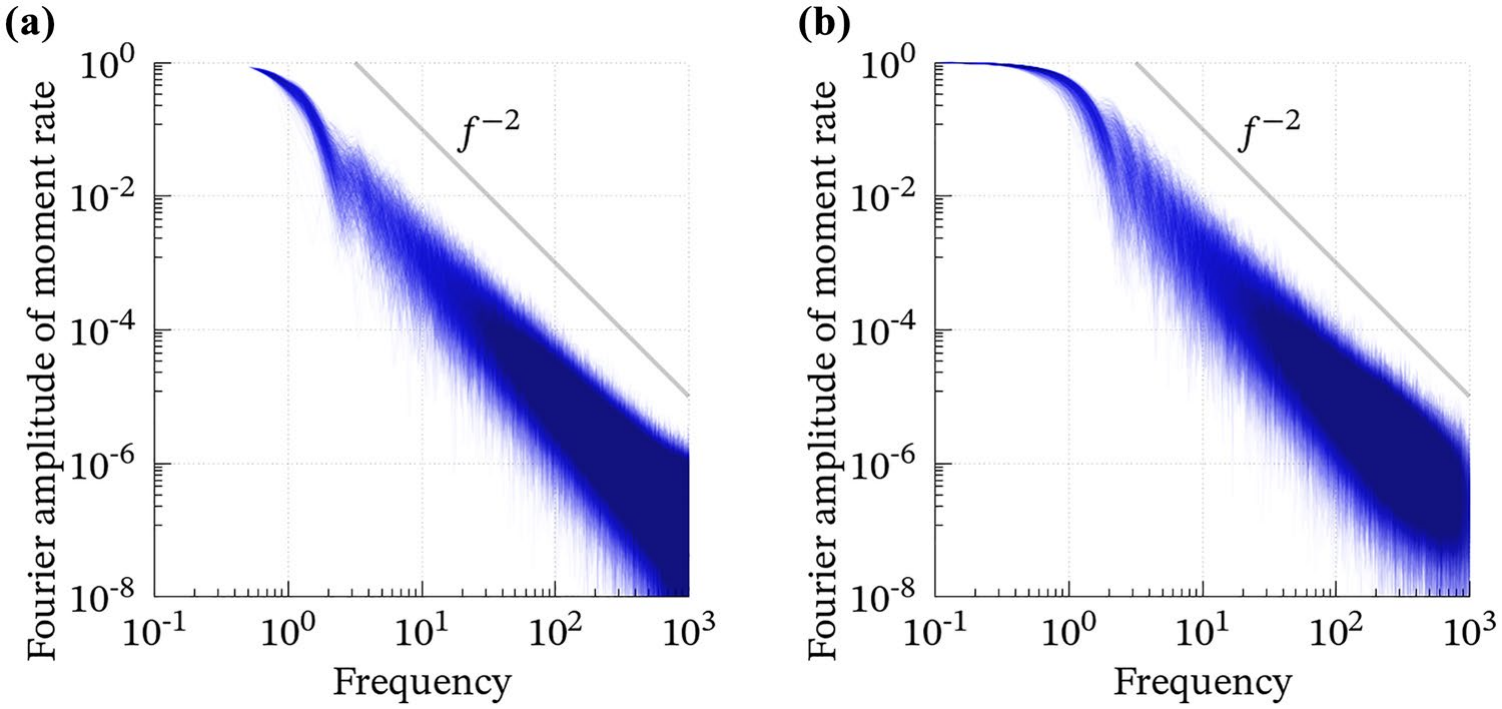
The normalized Fourier amplitude spectra of the convolutions plotted in Fig.2 for (**a**) case A and (**b**) case B.

The 1000 convolutions dominantly satisfy EL1 (Fig. 2), whereas the case B shows more variation. Although their average (i.e., the dense band in Fig. 2) seems like a smooth, bell-shaped, and compactly supported function that has been widely used as an input of wavefield simulations known as the Herrmann, Cosine, or Küpper wavelet functions^27^, individual cases are more complicated and sometimes skewed and/or multimodal; see Supporting Figures S.1 and S.2. Simultaneously, the time integration (Fig. 3) and Fourier amplitude spectra (Fig. 4) reproduce EL2 and EL3, respectively. EL4 is almost surely satisfied, as discussed in the previous section. Hence, we conclude that the convolution of two Bessel processes stochastically fulfills EL1–EL4.

## Discussion

### Physical meaning of the convolution

Here, we interpret the physical meaning of the convolution of two Bessel processes. In a simplified view, the convolution of two functions with the duration comparable to the whole event resembles the classical kinematic modeling of the source features as the convolution of two processes having two characteristic time scales: the rise time and the rupture time. However, the relationships between these functions and quantities like stress drop and fault impedance have to be investigated to interpret the presented formalism in terms of rupture dynamics.

As mentioned in “Introduction”, EL2 holds except for great earthquakes so that the fault width is sufficiently smaller than the thickness of seismogenic layer. If $M_0 \propto t^2$ holds for great earthquakes instead of EL2, the average shape of normalized STFs should be close to a triangle^9^, which is obviously different from STFs in Fig. 3. Thus, in the following, we consider a finite 2-D flat fault surface $\Gamma$ embedded in an infinite 3-D space. A general case without the above assumption for considering scalings of great earthquakes is our future task. We define two convolutions: “$*$” as only in time and “${\tilde{*}}$” as in on-fault position and time. In the case of a finite fault, we assume that the stress drop rate, ${\dot{\sigma }}(\varvec{x}, t)$ for the on-fault position $\varvec{x} \in \Gamma$, can be represented as:

6 $$\begin{aligned} {\dot{\sigma }}(\varvec{x}, t) = -\left( V {\tilde{*}} {\dot{Z}}\right) (\varvec{x}, t), \end{aligned}$$

where $V(\varvec{x}, t)$ is the slip rate distribution again and $Z(\varvec{x}, t)$ is the fault impedance defined as the ratio of stress to slip rate in the space-time Fourier domain^16,17^. If the surrounding area is an elastic body, *Z* in the space-time Fourier domain can be derived from linear elasticity as Eq. (20) of Andrews^16^ (see Supporting Information for details). However, we consider a stochastic process in which *Z* includes a non-deterministic property. Equation (6) represents the stress rate (i.e., Neumann condition) based on the displacement discontinuity (i.e., Dirichlet condition) along a finite fault; thus, *Z* is mathematically called a Dirichlet-to-Neumann operator. Here, we assume that there exists a Neumann-to-Dirichlet operator ${\dot{Z}}^{-1}$, whose support is $\Gamma$, satisfying:

7 $$\begin{aligned} V(\varvec{x}, t) = -\left( {\dot{\sigma }} {\tilde{*}} {\dot{Z}}^{-1} \right) (\varvec{x}, t). \end{aligned}$$

Furthermore, the Fourier transform with respect to position ($\displaystyle \int _\Gamma e^{2 \pi i \varvec{k}\cdot \varvec{x}} d\varvec{x}$, where $\varvec{k}$ is a two dimensional wavenumber) yields:

8 $$\begin{aligned} V(\varvec{k}, t) = -\left( {\dot{\sigma }}(\varvec{k}, \bullet ) *{\dot{Z}}^{-1}(\varvec{k}, \bullet ) \right) (t). \end{aligned}$$

As the limit $\varvec{k} \rightarrow 0$ is equivalent to the integration in space $\left( \displaystyle \lim_{\varvec{k} \rightarrow 0} \displaystyle \int _\Gamma e^{2 \pi i \varvec{k}\cdot \varvec{x}} d\varvec{x} = \displaystyle \int _\Gamma d\varvec{x}\right)$, Eq. (8) results in

9 $$\begin{aligned} {\overline{V}}(t) = \mu ^{-1} {\dot{M}}(t) = -\left( \overline{{\dot{\sigma }}} *\overline{{\dot{Z}}^{-1}} \right) (t), \end{aligned}$$

where the overlines denote integration over $\Gamma$. Finally, Eq. (9) implies that EL1–4 are fulfilled if the stress rate, $\overline{{\dot{\sigma }}}(t)$, and Neumann-to-Dirichlet operator, $\overline{{\dot{Z}}^{-1}}$, when integrated over $\Gamma$, are Bessel processes.

**Figure 5:**
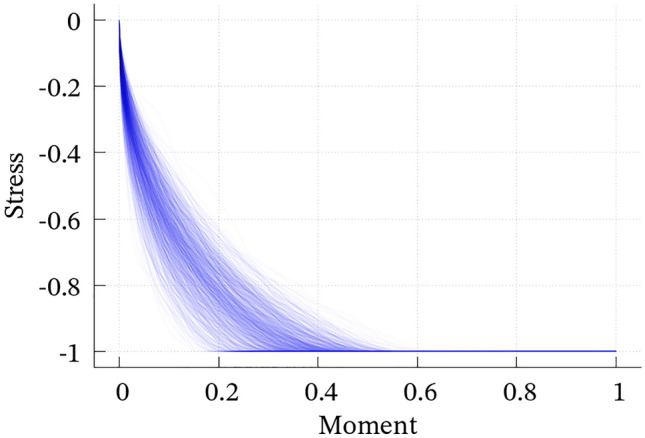
Normalized moment versus normalized stress drop assumed to be time-integration of a Bessel process for case A.

As $\sigma$ comprises stress *drop*, $-\overline{{\dot{\sigma }}}(t)$ is always non-negative and $-{\overline{\sigma }}(t)$ is a non-decreasing function from zero to its final value ($>0$). This property is naturally produced if $-\overline{{\dot{\sigma }}}(t)$ is a Bessel process. For the 1000 convolutions obtained in the previous section, we also calculate $- \displaystyle \int _0^t X_s^{(1)} \, ds$, where the duration of $X_s^{(1)}$ is shorter than that of $X_s^{(2)}$. By considering this quantity as ${\overline{\sigma }}(t)$, we confirm that the relationship between *M*(*t*) and ${\overline{\sigma }}(t)$ shows monotonic slip-weakening curves (Fig. 5). Therefore, the assumption that the stress drop rate is a Bessel process explains the natural weakening process of the on-fault stress change. In Fig. 5, the abscissa and ordinate mimic integrated slip and stress drop over the fault, respectively. This means that the characteristic slip weakening distance ranges from 20 to 50% of the final slip amount. Interestingly, this fraction is close to results based on observations. Mikumo et al.^28^ estimated the final slip amount ($D_\text {max}$) and slip weakening distance ($D_c$) for each subfault of the 2000 Tottori earthquake and concluded that $0.27 D_\text {max}< D_c < 0.56 D_\text {max}$ holds in almost subfaults. Although they revealed point-wise $D_c-D_\text {max}$ relations, their average over all subfaults also satisfies the same inequality (if $0.27 x_i< y_i < 0.56 x_i$ holds for each $i=1, \ldots , N$, also $\frac{0.27}{N} \sum _{i=1}^N x_i< \frac{1}{N} \sum _{i=1}^N y_i < \frac{0.56}{N} \sum _{i=1}^N x_i$ holds, where $\frac{1}{N} \sum _{i=1}^N x_i$ and $\frac{1}{N} \sum _{i=1}^N y_i$ are average values of $x_i$ and $y_i$, respectively).

As we consider Eqs. (1) and (5) with $b \sim 1$, our model consists of the drift term with negative feedback ($\frac{d-1}{2} \frac{dt}{X_t}$, where $\frac{d-1}{2} < 0$ and $X_t \ge 0$) and the diffusion term ($dB_t$). Especially, the amplitude of the drift term increases as the stress drop rate ($X_t$) decreases, which means that the drift term and diffusion term behave as the restoring force and stochastic driving force to stabilize and (possibly) evolve the stress drop rate, respectively, during the process.

To interpret the other assumption that the inverse fault impedance, $\overline{{\dot{Z}}^{-1}}$, is a random process is not straightforward, and we need more detailed discussion in the future work. When we calculate seismic waves, the Green functions are well modeled within the framework of linear elasticity. This might be because the Green functions depend on the medium between the fault and (usually) far-field observation points, where almost all of the region is an elastic body. However, the (inverse) fault impedance is a propagator among the on-fault positions traveling along the fault. In general, faults are segmented, bumpy, and surrounded by fractured rocks. Modeling such a complex system by assuming a flat fault may cause non-deterministic fluctuations due to scattering waves, as schematically illustrated by Aso et al.^19^. Therefore, this assumption is possible, even though it is difficult to directly observe.

In the numerical simulation, we restrict the ratio of the duration of $X_t^{(1)}$ and $X_t^{(2)}$ within tenfold. This is not only for EL3, as mentioned here, but also for another physical property. If $X_t^{(1)}$ is the stress drop rate, its duration should correspond to the duration of the most energetic faulting process, which is given by the fault length divided by the rupture speed. On the other hand, because $\overline{{\dot{Z}}^{-1}}(t) = X_t^{(2)}$ is based on the fault impedance, its duration must be equivalent to the time taken for the scattering wave to spread over the entire fault. This time is at least, or even a few times greater than, the fault length divided by the seismic wave speed. Therefore, the durations of $X_t^{(1)}$ and $X_t^{(2)}$ should have almost the same order, and $T_\text {min}/T_\text {max} = 0.5$ and 0.1 in our assumption might be two possible end members. We have to note that it is unclear whether the above discussion is applicable even for great earthquakes that EL2 may not hold as in the “Introduction”. For such cases, the point source approximation should be revised, and the different scaling property should be taken into account.

### Further mathematical and physical implication of the model

If the above discussion holds, the PDF of the first hitting time and the random variable in Fig. 1 represents the probability of termination and evolution of co-seismic stress drop on faults. Although this is not a pointwise but integrated distribution over the entire fault, the PDF gives us a constraint for forward and inverse modeling of stress drop evolution, which might be a significant implication of this model to fault dynamics.

Moreover, the mathematical structure of the system we considered would tell us more about fault dynamics. To satisfy EL1 and EL4, $X_t$ must be non-negative, and its duration (i.e., first hitting time) must follow some power law like Eq. (3). Other major types of SDE, including the Wiener process, Ornstein-Uhlenbeck process, and Cox-Ingersoll-Ross process, do not show the properties because they do not have any termination mechanism therein. Therefore, a model with other types of SDE^22^ is inherantly unable to explain EL1 and EL4, and the Bessel process is a prime candidate for our purpose. The squared Bessel process is another candidate, but it is essentially the same as the Bessel process because its square root is a solution of Eq. (1)^29^. As discussed in the previous subsection, the drift term of Eq. (1) may be related to a stress-rate-weakening property of friction if $X_t$ is stress drop rate. A relationship between the rate of frictional strength and rate of stress was suggested in an extended framework of rate- and state-dependent friction^30^. Although the previous friction model^30^ included a linear relation of them, our model may also suggest the possibility that the rate of stress change is a considerable quantity for the fault friction.

Finally, we discuss the mathematical potential of the present model to explain more various STFs. The fall-off rate of observed spectra has been modeled as $\omega ^{-p}$, and *p* may slightly deviate from 2^10,11^. In the present model, $p=2$ holds as explained in Mathematical modeling (i.e., $\omega ^{-1} \times \omega ^{-1} = \omega ^{-2}$). Thus, instead of the Bessel process, we have to consider some stochastic process that deviates from $\omega ^{-1}$ spectrum if $p \ne 2$ holds. The Brownian-like noise with such spectrum is called the fractional Brownian noise^31^, and The Bessel process with the fractional Brownian noise is called the fractional Bessel process^32^. Therefore, we may model $\omega ^{-p}$ spectrum with $p \ne 2$ by using the fractional Bessel process instead of Eq. (1).

## Conclusions and outlooks

Here we demonstrated that the four empirical laws on STFs, or moment-rate functions, can be reproduced by modeling STFs as the convolution of two Bessel processes with almost the same order of duration. Although many theoretical models have been suggested to explain some of EL1–4 as written in the “Introduction”, the advantage of the present model is that the model shows all properties of EL1–4, stochastic fluctuation as seen in observed STFs, and slip-weakening fault dynamics. In terms of fault dynamics, given the complexity of the geometry and surroundings of the faults, this result is comprehensible if both the stress drop rate and the inverse fault impedance follow a Bessel process.

Among the four laws, we should investigate the dominance of unimodality more precisely and statistically. As in Supporting figures, unimodal distributions dominate in both cases A and B, but the fraction of bimodal or multimodal distributions seems relatively more significant in case B, which means that the number of prominent peaks may depend on the ratio of the first hitting times. In a database of observed STFs, 80% of them are unimodal, and others are bimodal or multimodal, which was declared by Dynamic Time Warping method that is an application from speech recognition^3^. Therefore, we also should employ the Dynamic Time Warping method and gather statistics of the peaks in the numerical STFs to compare our results to the observation.

In some numerical models, spatial and fractal heterogeneity of stress drop^33^, fracture energy^18^, or fault geometry^34^ have been introduced. In the present model, however, we do not consider such spatial distributions, and our interpretation is that the model is equivalent to a point source as we take the limit $\varvec{k} \rightarrow 0$. Therefore, the effects of the above heterogeneities might be integrated and appear as the stochastic term in Eq. (1). Thanks to this simplification, we succeeded in constructing the model within a framework of SDE. Nevertheless, the present model still has extensibility for considering spatial heterogeneity. As in the previous subsection, we may be able to consider $\omega ^{-p}$ ($p \ne 2$) spectrum with the fractional Bessel process. Theoretically, the spectral fall-off rate *p* is related to the heterogeneity of slip distribution in space^35^, which implies that we could model the spatial heterogeneity via the extended SDE model.

Even in the limit of point source approximation, the present model has applicability to scientific and engineering studies on strong ground motions. One way to numerically simulate strong ground motions is to compute the convolution of an STF and the Green function. In the calculation, a few point sources that have $\omega ^{-2}$ spectrum are sufficient to reproduce dominant parts of far-field seismic records deterministically^36^. We can obtain numerous inputs to a strong ground motion simulation and gather statistics of the simulated seismograms. Moreover, even without numerous numerical simulations, we can investigate the statistical properties of a stochastic process if the PDF of the random variable at any time is available as in Fig. 1. Thus, it should be possible to calculate some statistical properties of simulated strong ground motions at low computational costs.

## Supplementary Information

Supplementary Information 1.

Supplementary Information 2.
