# Supplementary material for: Source time functions of earthquakes based on a stochastic differential equation
Source: Sci Rep. 2022 Mar 10;12:3936. doi: 10.1038/s41598-022-07873-2 (PMC8913777; doi:10.1038/s41598-022-07873-2)
Supplement: Supplementary file 1 — Supplementary Information 1. [file 41598_2022_7873_MOESM1_ESM.pdf]

Supporting Figures for

Source time functions of earthquakes based on a stochastic differential equation

Shiro Hirano[1,*]

[1]Department of Physical Science, College of Science and Engineering, Ritsumeikan University, 1-1-1, Nojihigashi, Kusatsu, Shiga, 525-8577, Japan.
[*]s-hrn@fc.ritsumei.ac.jp

ABSTRACT

Individual results of the 1,000 convolutions are shown for case A (Fig.S.1) and case B (Fig.S.2). Five results are plotted in different colors for each.

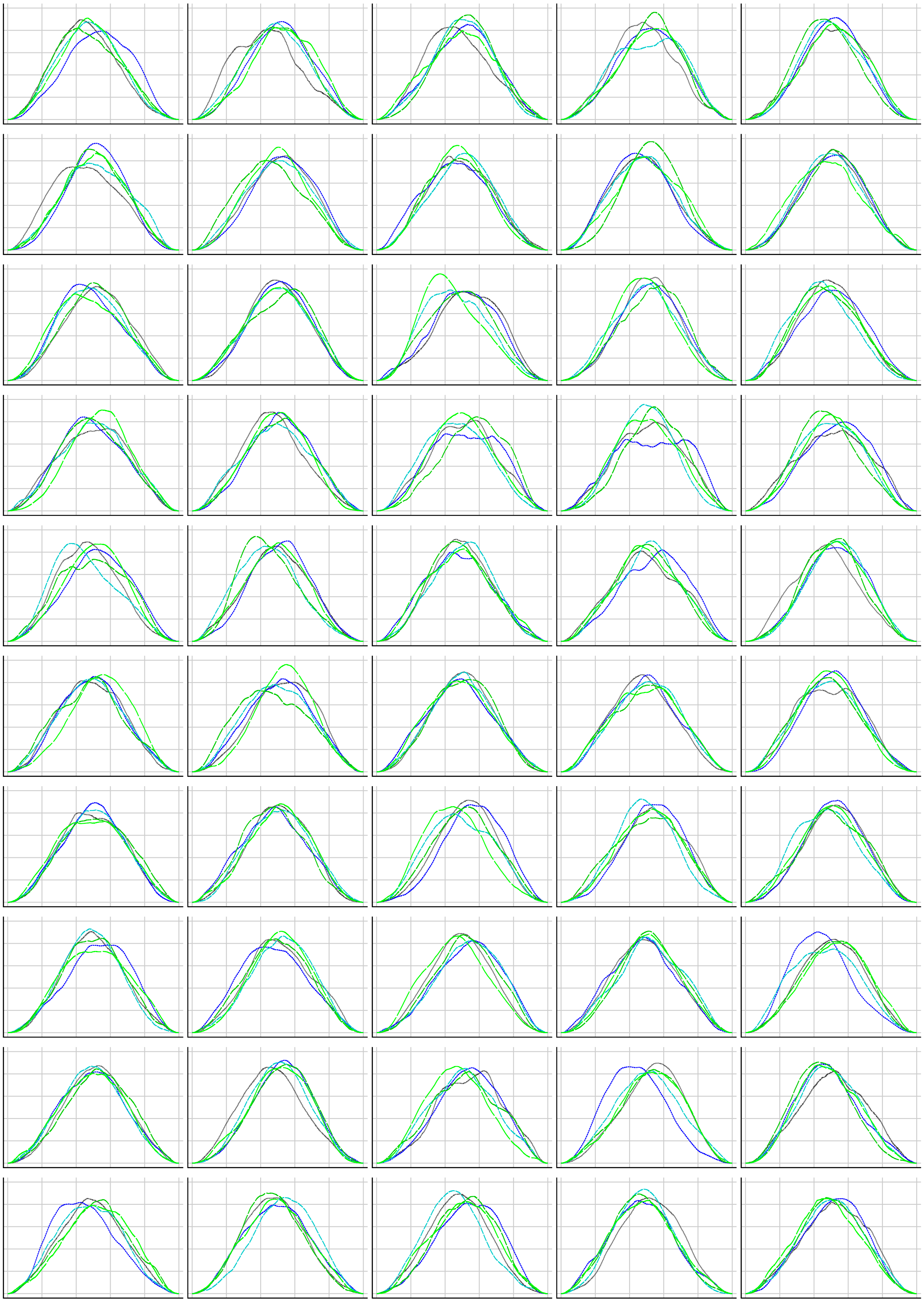

Figure S.1. The 1,000 convolusions (4 pages $\times$ 10 raws $\times$ 5 columns $\times$ 5 per each) for case A. The abscissae and ordinates are normalized by each duration and total moment, respectively (conti.)

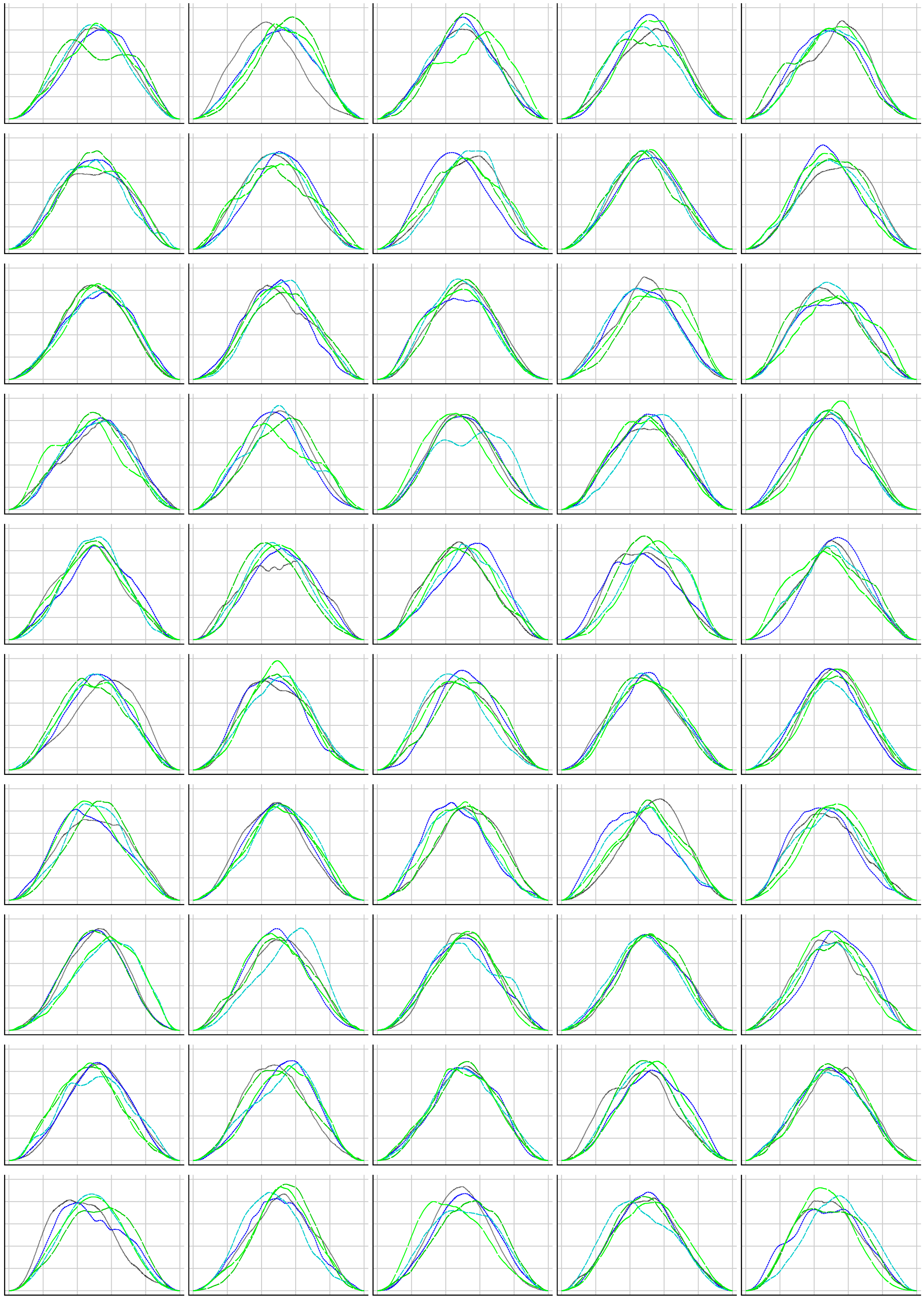

Figure S.1. (conti.)

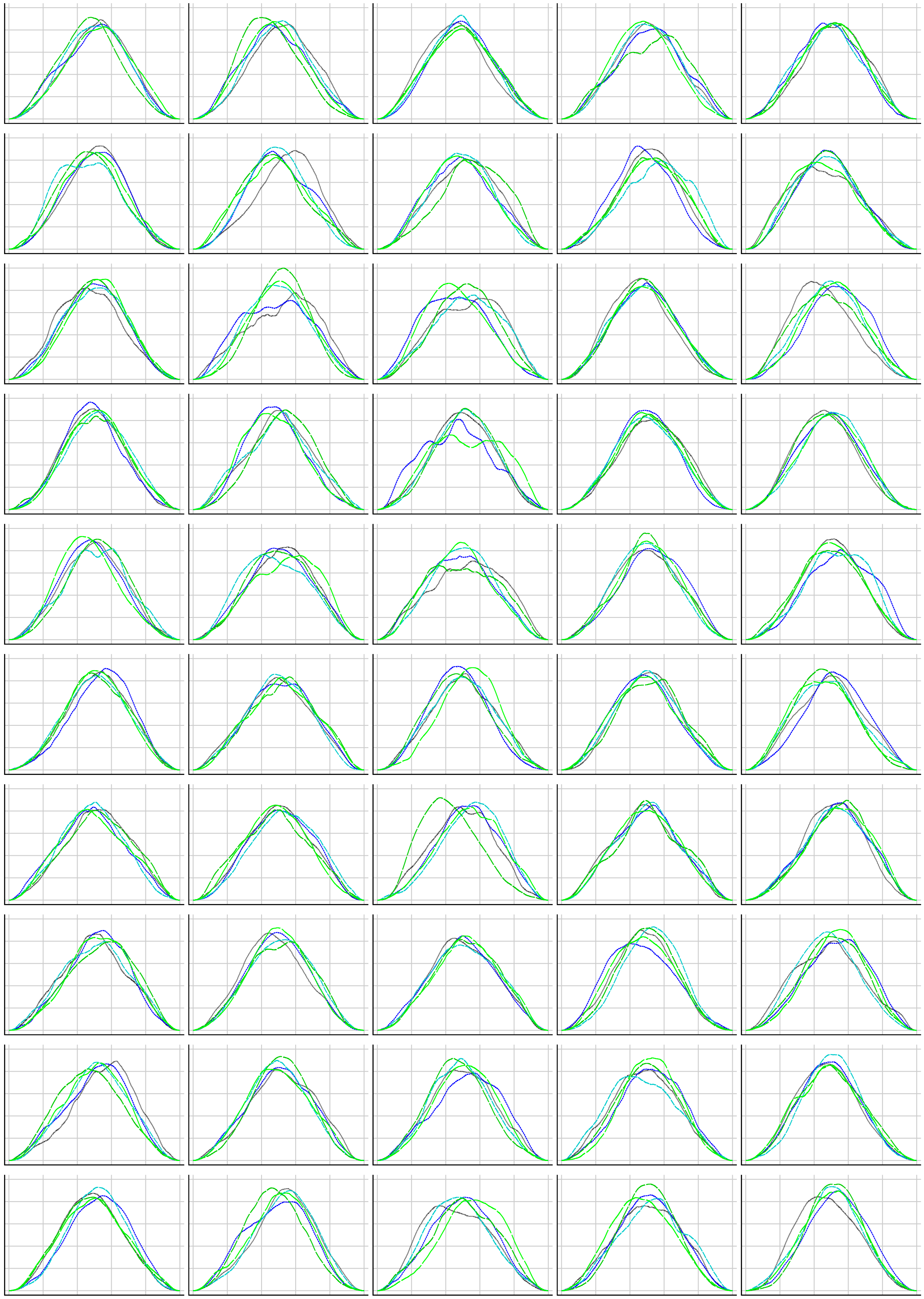

Figure S.1. (conti.)

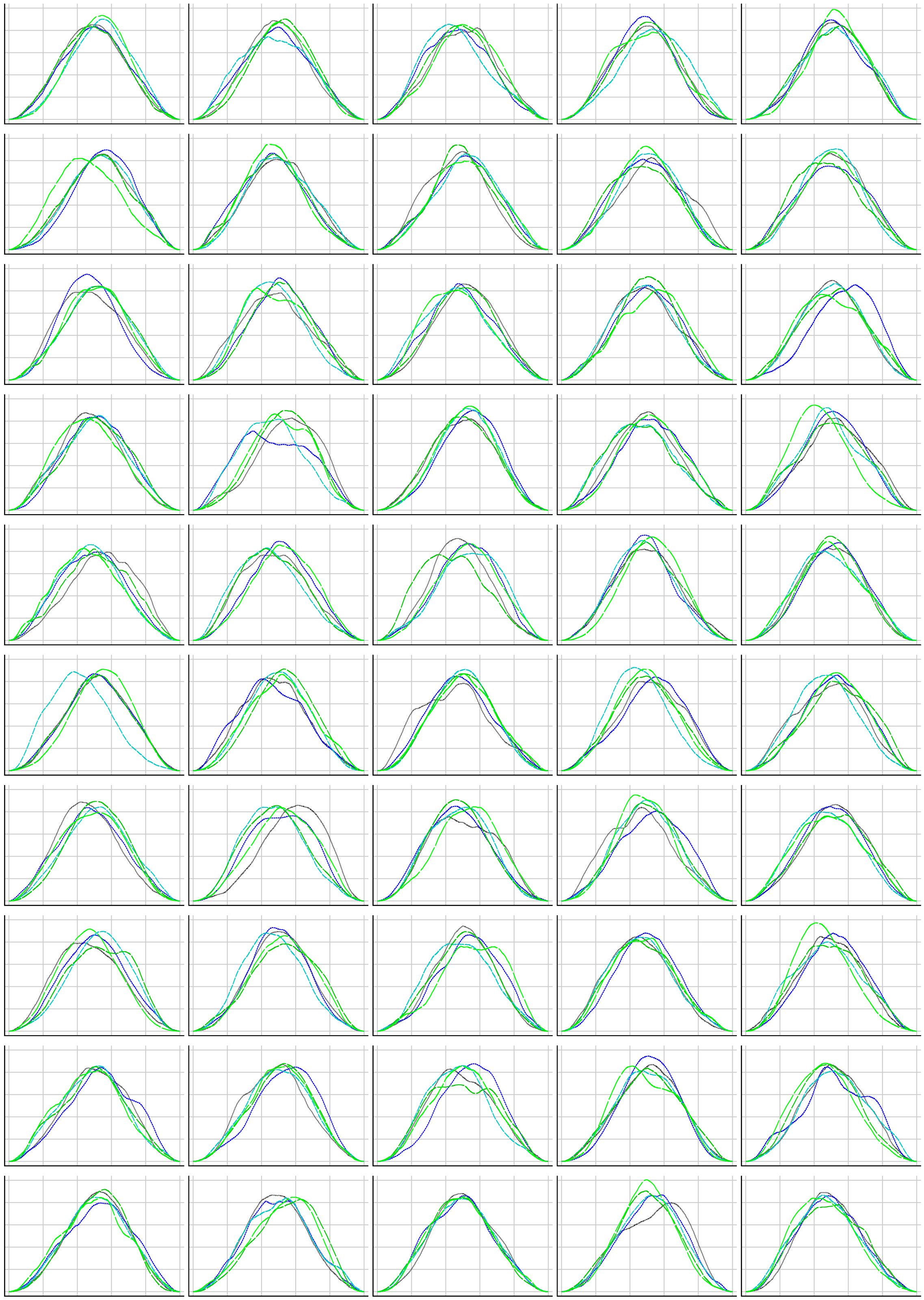

Figure S.1. (end.)

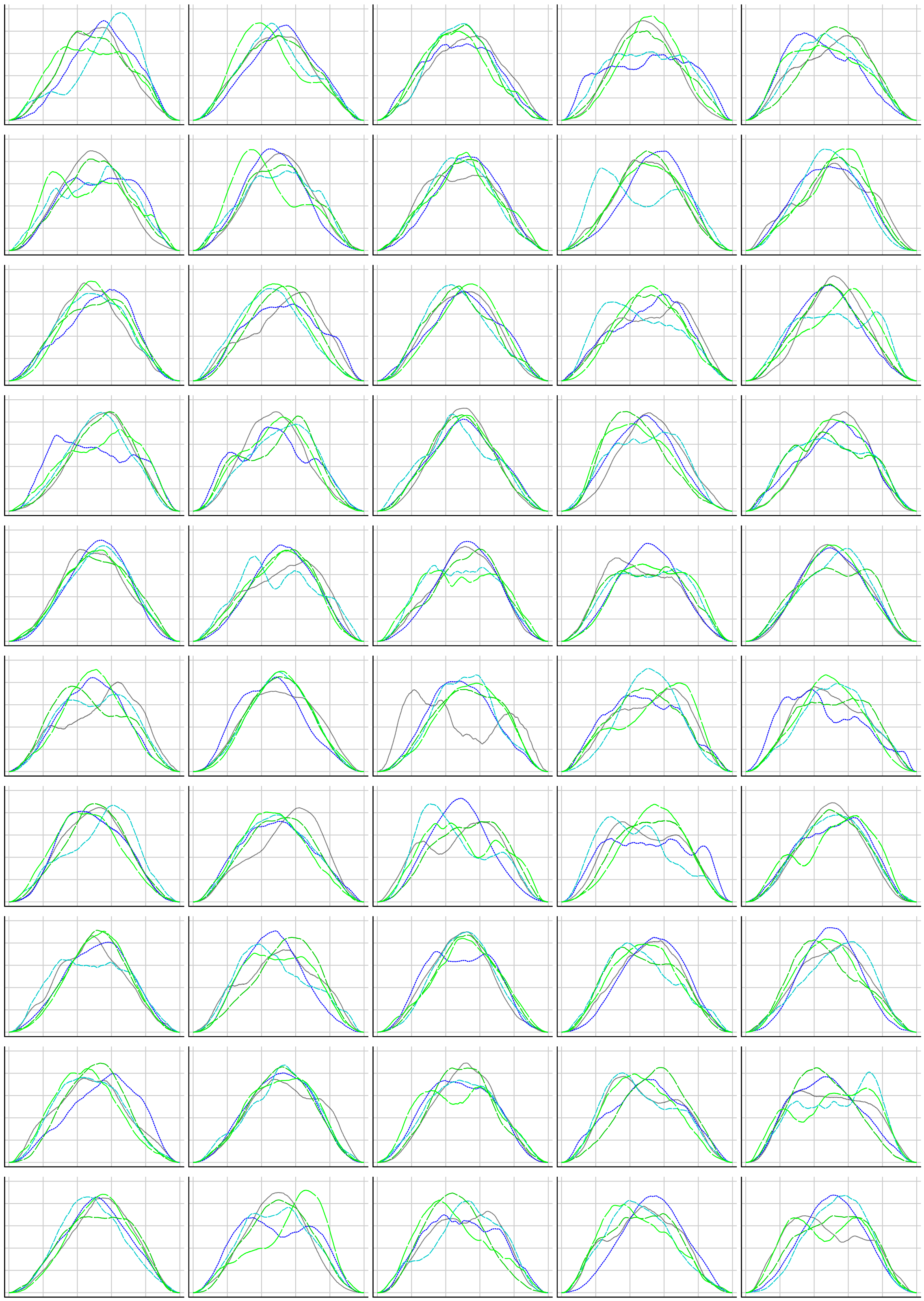

Figure S.2. The same plots as Fig.S.1 for case B. (conti.)

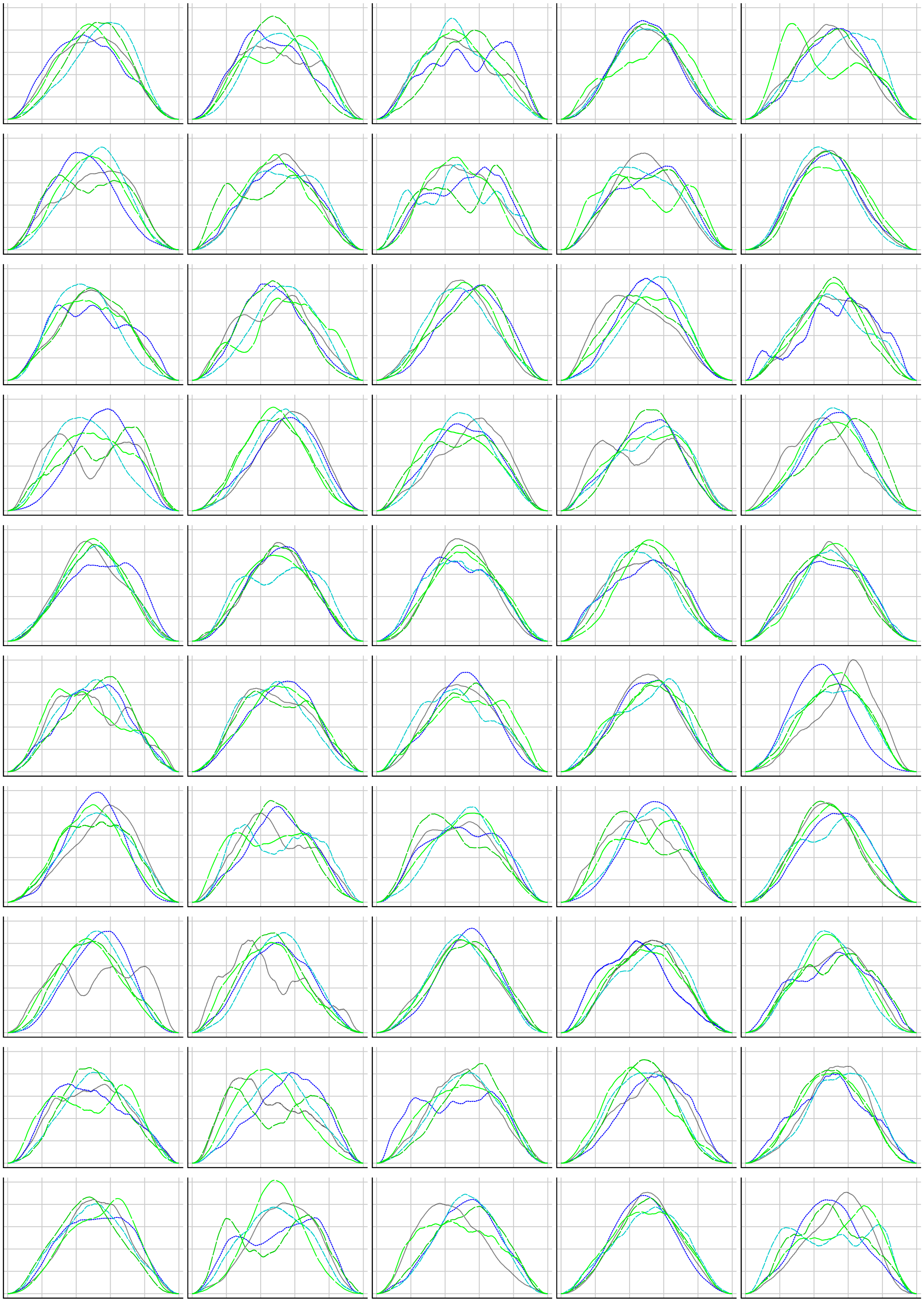

Figure S.2. (conti.)

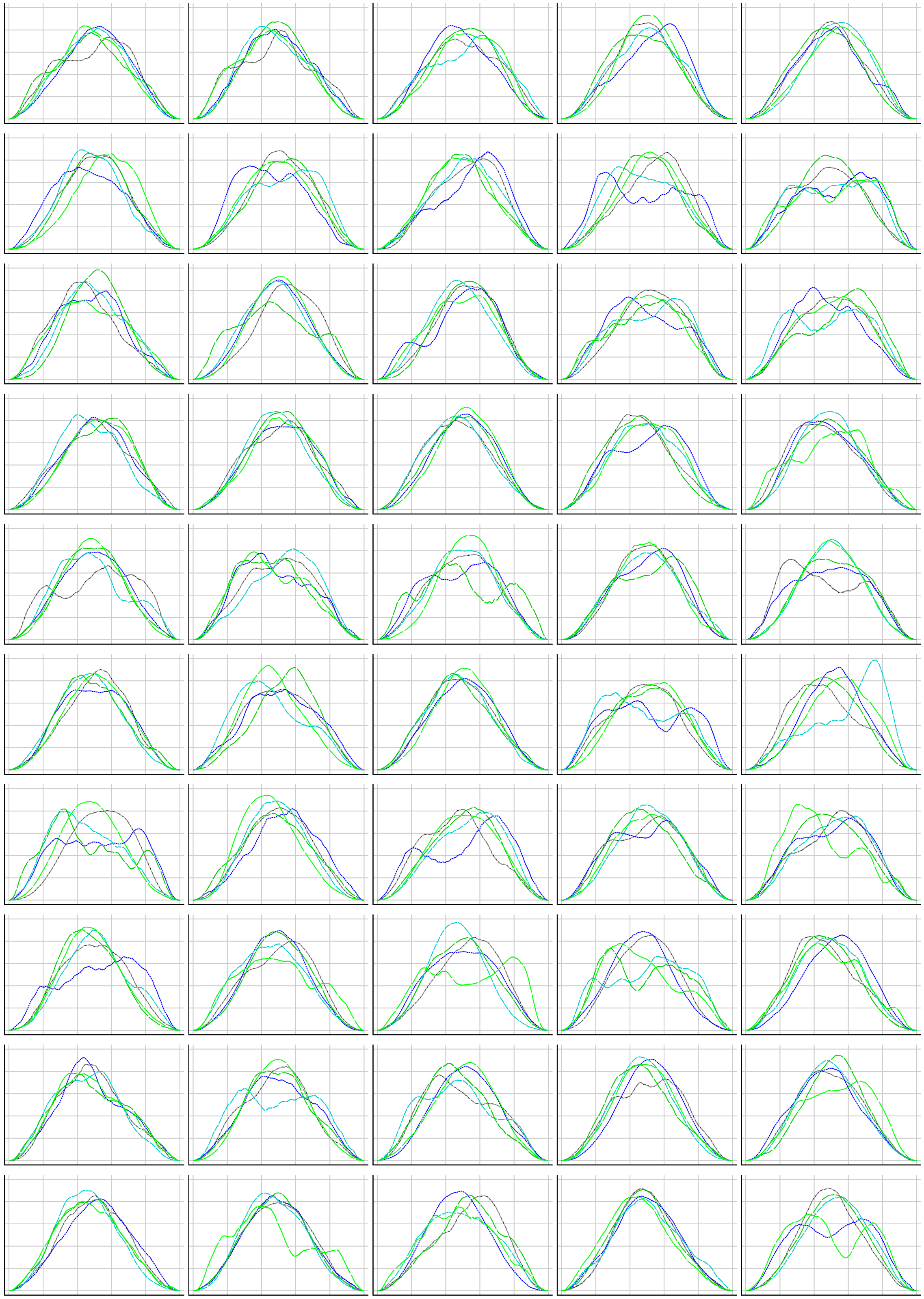

Figure S.2. (conti.)

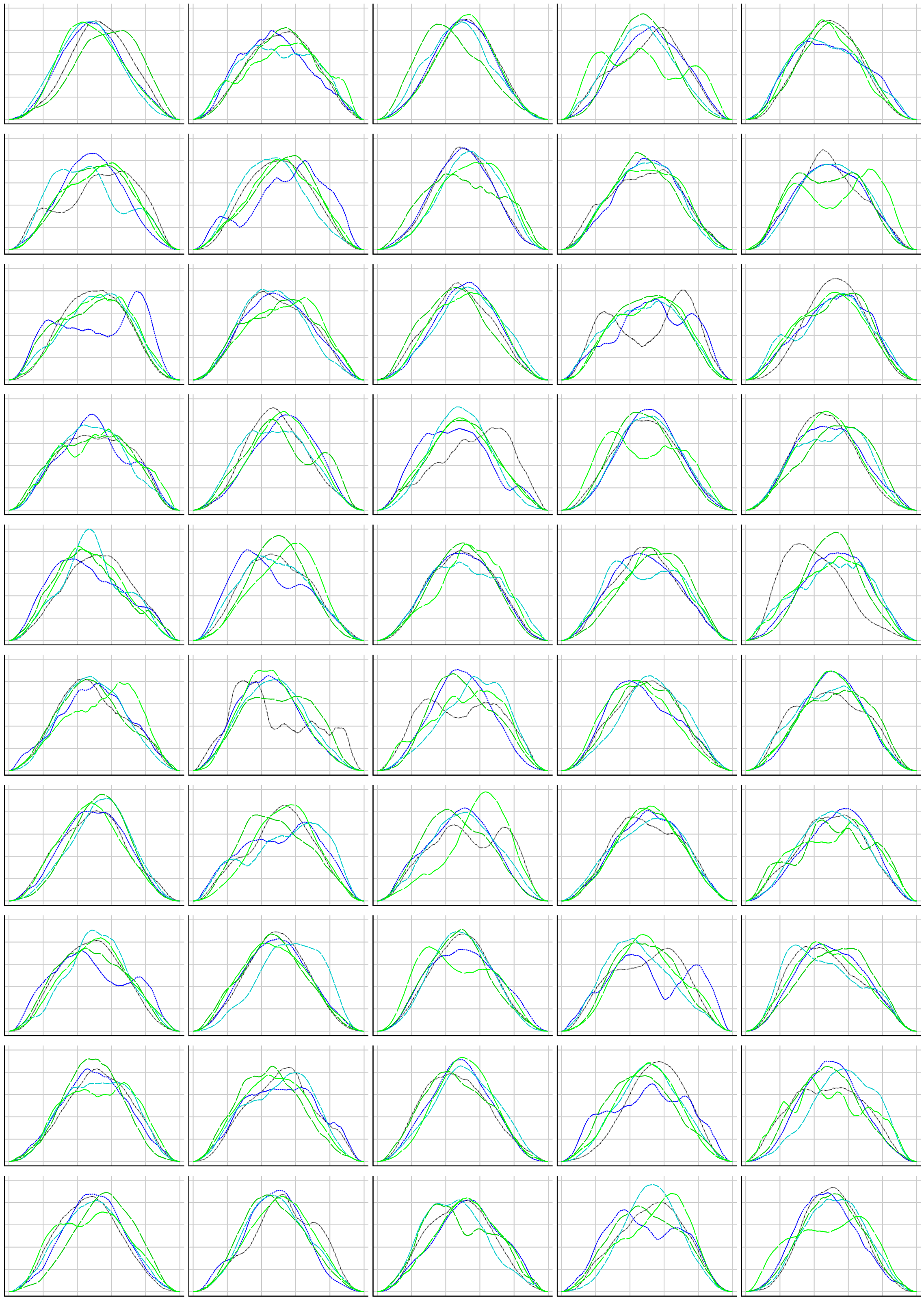

Figure S.2. (end.)
