# Supplementary material for: Source time functions of earthquakes based on a stochastic differential equation
Source: Sci Rep. 2022 Mar 10;12:3936. doi: 10.1038/s41598-022-07873-2 (PMC8913777; doi:10.1038/s41598-022-07873-2)
Supplement: Supplementary file 2 — Supplementary Information 2. [file 41598_2022_7873_MOESM2_ESM.pdf]

Supporting Information for

Source time functions of earthquakes based on a stochastic differential equation

Shiro Hirano[1,*]

[1]Department of Physical Science, College of Science and Engineering, Ritsumeikan University, 1-1-1, Nojihigashi, Kusatsu, Shiga, 525-8577, Japan.
[*]s-hrn@fc.ritsumei.ac.jp

ABSTRACT

The fault impedance

The fault impedance in the Discussion section was introduced by Andrews[1] and defined as the ratio of stress drop to slip rate in the space-time Fourier domain with the 2-D wavenumber vector (k_1, k_2) and the angular frequency ω. He considered a flat fault embedded in an infinite, homogeneous, and isotropic 3-D elastic body with the P-wave velocity α, S-wave velocity β, and rigidity μ. His eq.(20) was

$$Z = -\frac{\mu}{2\beta}\left\{4\beta^3\frac{\zeta_1^2}{\zeta^2}\left[\zeta^2\eta_P + \left(\zeta^2 - \frac{1}{2\beta^2}\right)^2\eta_S^{-1}\right] + \frac{\zeta_2^2}{\zeta^2}\beta\eta_S\right\}, \tag{20}$$

where $(\zeta_1, \zeta_2) = \left(\frac{k_1}{\omega}, \frac{k_2}{\omega}\right)$ is the slowness vector with the length of $\zeta = \sqrt{\zeta_1^2 + \zeta_2^2}$. η_P and η_S are defined as $\eta_P = \sqrt{\alpha^{-2} - \zeta^2}$ and $\eta_S = \sqrt{\beta^{-2} - \zeta^2}$.

References

1. Andrews, D. J. Fault impedance and earthquake energy in the fourier transform domain. *Bull. Seismol. Soc. Am.* **70**, 1683–1698, DOI: 10.1785/bssa0700051683 (1980).
